# Self-Organized Multi-Camera Network for a Fast and Easy Deployment of Ubiquitous Robots in Unknown Environments

**DOI:** 10.3390/s130100426

**Published:** 2012-12-27

**Authors:** Adrián Canedo-Rodriguez, Roberto Iglesias, Carlos V. Regueiro, Victor Alvarez-Santos, Xose Manuel Pardo

**Affiliations:** 1 CITIUS, University of Santiago de Compostela, 15782 Santiago de Compostela, Spain; E-Mails: roberto.iglesias.rodriguez@usc.es (R.I.); victor.alvarez@usc.es (V.A.-S.); xose.pardo@usc.es (X.M.P.); 2 Department of Electronics and Systems, University of A Coruña, 15071 A Coruña, Spain; E-Mail: cvazquez@udc.es

**Keywords:** robot deployment, robot detection and tracking, multi-camera networks, ambient intelligence, ubiquitous robots

## Abstract

To bring cutting edge robotics from research centres to social environments, the robotics community must start providing affordable solutions: the costs must be reduced and the quality and usefulness of the robot services must be enhanced. Unfortunately, nowadays the deployment of robots and the adaptation of their services to new environments are tasks that usually require several days of expert work. With this in view, we present a multi-agent system made up of intelligent cameras and autonomous robots, which is easy and fast to deploy in different environments. The cameras will enhance the robot perceptions and allow them to react to situations that require their services. Additionally, the cameras will support the movement of the robots. This will enable our robots to navigate even when there are not maps available. The deployment of our system does not require expertise and can be done in a short period of time, since neither software nor hardware tuning is needed. Every system task is automatic, distributed and based on self-organization processes. Our system is scalable, robust, and flexible to the environment. We carried out several real world experiments, which show the good performance of our proposal.

## 1. Introduction

In the following decades, personal service robots are expected to become part of our everyday life, either as assistants, house appliances, collaborating with the care of the elderly, *etc.* In this regard, personal service robots are gaining momentum with a first generation that performs limited yet useful tasks for humans in human environments, enabled by progresses in robotics' core fields such as computer vision, navigation, or machine learning. In parallel, hardware elements like processors, memories, sensors, and motors have been continuously improving, while their price has been dropping. This makes roboticists positive about the possibility of building quality robots available to the vast majority of society. Moreover, since some companies are already investing in business models such as robot renting, getting robots to work in places like museums, conferences, or shopping centres will become more affordable. Hence, it is not surprising to see how the market of personal robots has increased over the last years; expectations are to even surpass the current trend [1]. Even more, according to our experience, more and more research groups are being requested to take their robots to social events (e.g., public demonstrations). In our opinion, all of this reflects the increasing interest of society for robots that assist, educate, or entertain in social spaces. At this point, it is paramount to start providing affordable solutions to answer to society's demand.

Apart from the core problems that remain to be solved (SLAM, online learning, human-robot interaction, *etc.*), there are two problems that are restraining this first generation of robots to get out of the research centres: (1) the cost of the deployment of robotic systems in unknown environments, and (2) the poor perception of the users about the quality of the services provided by the robots. We call “deployment” to all that must be carried out to get a robot operating in a new environment. Ideally, this deployment should be fast and easy, but in practice it requires experts to adapt both the hardware and the software of the robotic system to the environment. This includes programming “*ad hoc*” controllers, calibrating the robot sensors, gathering knowledge about the environment (e.g., metric maps), *etc.* This adaptation is not trivial and requires several days of work in most cases, making the process inefficient and costly. Instead, we believe that the deployment must be as automatic as possible, prioritizing online adaptation and learning over pre-tuned behaviours, knowledge injection, and manual tuning in general. On the other hand, if we really want the robots to be considered useful, they must provide services of quality. In this sense, it would be very useful if robots could show initiative, offering services that anticipate users' needs.

Unfortunately, most service robots carry out all the deliberation and action selection on-board, based only on their own perceptions. This is highly restrictive, since these robots are only able to react to events that occur in their surroundings. Opposed to this philosophy, a new paradigm called ubiquitous robotics [2] proposes to distribute intelligence, perception and action components amongst a set of networked devices (laptops, smart-phones, sensors…) to build up an “ubiquitous space”. Within this paradigm, for instance, a robot can perceive users' needs anywhere in the ubiquitous space, regardless of where the robot is.

In this paper, we propose to combine technologies from ubiquitous computing, ambient intelligence, and robotics, in an attempt to get service robots to work in different environments. The deployment of our system is fast and easy, since it does not require any tuning, and every task is designed to be automatic. Basically, we propose to build an intelligent space that consists of a multi-agent distributed network of intelligent cameras and autonomous robots. The cameras are spread out on the environment, detecting situations that might require the presence of the robots, informing them about these situations, and also supporting their movement in the environment. The robots, on the other hand, navigate safely within this space towards the areas where these situations happen.

In Section 2 we present a review of the previous works on service robots, focusing on their capability to be deployed fast and easy. In Section 3 we provide a general description of the system that we propose. In Section 4 we describe the general requirements of our system. In Section 5 we present the main tasks performed by our system. In Section 6 we present several experiments that validate our proposals. Finally, Section 7 summarizes the main conclusions, and our future lines of work.

## 2. Previous Work

Over the last decades, there has been a big effort to bring service robots to social events. A great number of the most remarkable examples are information and guide robots, such as Rhino (1995) [3], Minerva (1999) [4], Robox (2003) [5], Tourbot and Webfair (2005) [6], and Urbano (2008) [7]. Apart from the evolution on the robots' quality, their deployment time decreased down to an acceptable point. For instance, Rhino and Minerva originally required 180 and 30 days of installation, while Tourbot and Webfair could be deployed in less than two days [6]. Following this trend, Urbano required less than an hour for a basic installation (map building for localization) [7]. Regretfully, this has not been the case regarding ubiquitous robotics, where the major proposals so far did not tackle the problem of the easy and fast robot deployment.

Lee *et al.* (Hashimoto Labs.) were pioneers in proposing the concept of intelligent spaces as rooms or areas equipped with sensors that perceive and understand what is happening in them and that can perform several tasks for humans [8]. They proposed a system of distributed sensors (typically cameras) with processing and computing capabilities. With this system, they were able to support robots' navigation [9,10] in small spaces (two cameras in less than 30 square meters). Similarly, the MEPHISTO project [11,12] proposed to build 3D models of the environment from the images of highly-coupled cameras. Then, they utilized these models for path planning and robot navigation. Their experiments were performed in a small space (a building's hall) with four overlapped cameras. Finally, the Electronics Department of the University of Alcalá (Spain) has proposed several approaches for 3D robot localization and tracking using a single camera [13] or a camera ring [14].

Moreover, they have proposed a navigation control strategy that uses rings of cameras [15], which was tested in a 45 *m*^2^ laboratory with four cameras. All of these works have demonstrated the feasibility of robot navigation supported by external cameras. Regretfully, all of them have been designed and validated to work in small places with a great number of sensors, which compromises their scalability and cost. Furthermore, except for the Hashimoto Labs' ones, the rest of the works are not focused on the decentralized control of the robot, but mainly on its 3D localization by using the information from the cameras. Moreover, they rely on a centralized processing of the information, and wired communications, making the scalability even harder. We believe that these problems are not due to limitations of the proposals, but to the fact that the easy and fast deployment of the system was not considered as an objective.

A different concept is explored in the PEIS Ecology (Ecology of Physically Embedded Intelligent Systems) [16,17], which distributes the sensing and actuation capabilities of robots within a device network, such as a domotic home would do. They focus on high level tasks, such as in designing a framework to integrate a great number of heterogeneous devices and functionalities [18] (cooperative work, cooperative perception, cooperative re-configuration upon failure or environment changes…). However, this project does not tackle low level tasks critical for our purposes, such as robot navigation or path planning.

The closest works to our philosophy are the Japan NRS project and the URUS project. The NRS project focuses on user-friendly interaction between humans and networked environments. These environments consist of sensors for motorization and robots and other devices to offer information. On the line of our work, they demonstrated the use of their systems in large real field settings, such as science museums [19], shopping malls [20], or train stations [21], during long term exhibitions. However, a number of factors make these systems inadequate for our purposes. First, most communications are wired and all processing takes place in a central server, which compromises the systems' scalability. Second, their robots are not fully autonomous, since a human operator controls them in certain situations. Finally, they did not use video-cameras to sense the environment, which we intend to, since they can provide rich information of the environments and human activities. On the other hand, the URUS project (Ubiquitous networking Robotics in Urban Settings) [22] proposes a network of robots, sensors and other networked devices to perform different tasks (informative tasks, goods transportation, surveillance, *etc.*) in wide urban areas (experimental setup of 10,000 *m*^2^). The system has shown its feasibility to work in urban areas [23], but in the context of our problem, some of the proposals are not appropriate: for example, the system requires a 3D laser map of the environment prior to its deployment [23], the cameras are connected via Ethernet to a rack of servers that process their information [23], the control processes (e.g., task allocation) take place in a central station [24] rather than being distributed, *etc.* This makes clear that neither the efficiency of the deployment phase nor other characteristics such as the scalability and flexibility to introduce new elements in the system were considered (probably because the system is intended to operate always in the same urban area).

The projects above are the most representative in the context of our work. Even more, Sanfeliu *et al.* [25] described the last three projects (PEIS, NRS and URUS) as “the three major on-going projects in Network Robotic Systems” by the end of 2008, and to the best of our knowledge, this assertion is still valid. We consider that the described works do not target the problem of the efficient robot deployment in unknown environments. Thus, a solution to get robots out of the laboratories within reasonable times and costs is still to be proposed.

## 3. System Description

**Figure 1.**
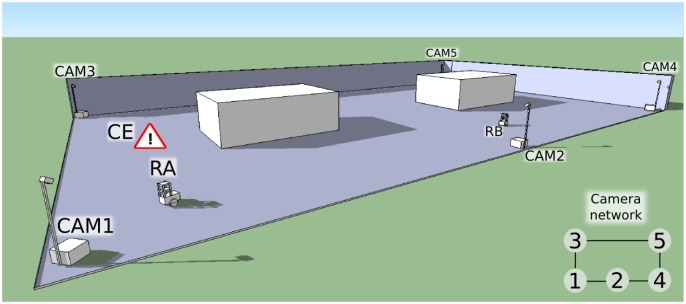
Example of the deployment of the multi-agent system: Camera-agent 3 (CAM3) is detecting a Call Event, while robot-agent A (RA) is being sighted by camera-agent 1 (CAM1) and robot-agent B (RB) by camera 2 (CAM2).

**Figure 2.**
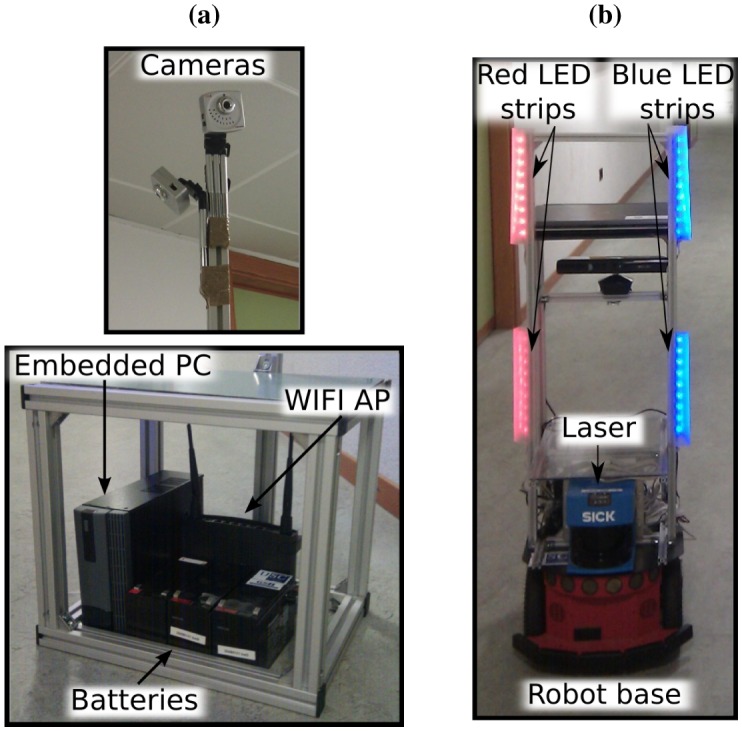
(**a**) Camera-agent: camera-agent's cameras (top), and camera-agent's box with embedded PC, batteries and WiFi AP (left-bottom). (**b**) Robot-agent, with its laser range finder and LED strips for identification from the cameras.

We aim at developing a system that provides a framework for the fast and easy deployment of ubiquitous robots in diverse environments. We propose a multi-agent system such as the one illustrated in Figure 1, which consists of two main elements: (a) an intelligent control system formed by camera-agents spread out on the environment (CAM1 to CAM5 in the figure), and (b) autonomous robots navigating on it (RA and RB, in the figure). Each camera-agent consists of an aluminium structure like that of Figure 1, which is easy to transport, deploy and pick up. This aluminium structure has two parts: A box and a mast attached to it. As it is shown in Figure 2(a), the box contains a processing unit, a WiFi Access Point and power supplies, while the mast holds one or more video cameras (one for each camera-agent). Regarding the robots, we work with Pioneer 3DX robots like the one in Figure 2(b). These robots are equipped with a laser scanner, sonar sensors, and a set of colour LED strips, which form patterns that can be recognized from the cameras, as we will explain in Section 5.4.

Our system is cost-effective for several reasons. First of all, the hardware cost of each physical camera-agent is 1, 300$ approximately, including: 3 cameras, 1 laptop, 1 WiFi AP, 4 batteries, and the aluminium structure. This cost is relatively low compared with the cost of each robot. Moreover, the cost can be even cheaper, for example by replacing the laptops with single-board computers. Therefore, our system offers the benefits of ubiquitous robotics, without shooting up the costs with respect to non-ubiquitous approaches [3,7]. Second, as we will see, each camera can support the robot navigation up to a distance of 20 meters approximately. This ensures the coverage of big areas with a low number of camera-agents, as opposed to other ubiquitous robotics works [9,11,15]. Finally, our system does not require any software development or hardware tuning in order to be deployed in a new environment. This in fact can represent a great cost reduction compared with other similar systems whose deployment is more complex, such as [23]. In addition, on the contrary to some of the aforementioned systems, we do not require the existence of a cable network, because the communications amongst our agents are lightweight enough to work with wireless networks.

## 4. System Requirements

The system must be easy and fast to deploy in different environments. In this sense, all the elements of our system must be easy to transport, to mount and, over all, to configure. On the other hand, the system must be scalable, meaning that the introduction or elimination of elements during operation should be easy and fast (no re-design and minimal re-configuration required). Therefore, the system will prioritize self-configuration over manual tuning, and it should not require previous knowledge of the environment (like metric maps). We would like to point out that we do not want to exclude SLAM solutions from our system; instead, we believe that we should combine several methods in order to achieve a robust solution to operate in real world environments. So far, we have avoided the use of maps for the sake of easy and fast deployment, but we plan to include SLAM techniques in the future to increase the robustness of our proposal [26]. Even in absence of this knowledge (metric maps), the system will be flexible to work in different environments, and robust to cope with uncontrolled and unpredictable conditions: People moving around, challenging illumination, furniture changes, *etc.* Finally, although it would be very helpful, it is usually the case that those people who are responsible for the events where the robot works do not allow modifications in the environment (e.g., place landmarks, change the illumination, move the furniture, lay cables, *etc.*).

Once deployed, our system will detect those situations that require the assistance of our robots, and it will enable our robots to navigate towards the areas where these situations are detected. The camera network will be in charge of detecting such situations (called Call Events, or CEs like in Figure 1). This will increase the range of action of our robots.

We would like to point out that the concept of Call Event can be accommodated to a wide range of situations. For example, Call Events may be triggered by users asking explicitly for assistance (e.g., by waving in front of a camera). Even more, Call Events can be activated even if no user triggers them explicitly, for instance taking into account the behaviour of the people in the environment. This is very interesting, because it would enable the robots to anticipate to the users' needs [27]. For example, in the context of a museum tour-guide robot, our cameras could detect groups of people that are staying still at the entrance of the museum, infer that they may need assistance, and send the robot towards them. This will indeed be perceived by the users as a pro-active behaviour.

## 5. System Tasks

**Figure 3.**
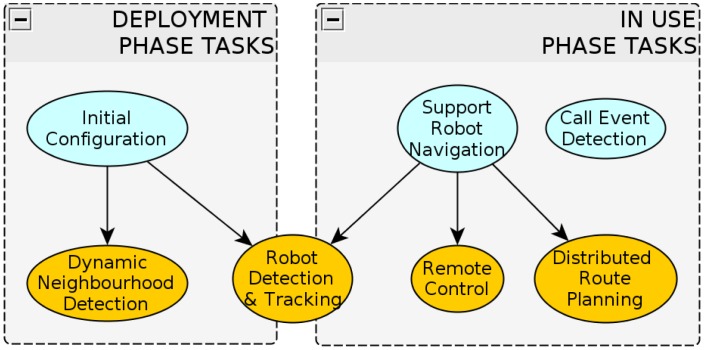
Camera tasks during the deployment and in use phases.

To set our system working and accomplishing the requirements described in Section 4, we need to consider two separate and important phases: the “deployment” phase and the “in use” phase. Each phase requires different tasks, represented in Figure 3. All the tasks of the system are coordinated in a self-organized manner [28]. We must bear in mind that there are several definitions of self-organization, some of which imply the existence of emergence, while some others do not [29]. We consider our system self-organized because coordination arises from local interactions among independent agents. Moreover, the system is fully distributed, since there is no hierarchy or centralization in either control or knowledge storage. However, we would like to point out that there are no emergent processes in our system.

On the “deployment phase”, the camera agents carry out an initial configuration. This consists of establishing neighbourhood relationships among them (dynamic neighbourhood detection), by detecting simultaneous events in their Fields of View (FOVs). Since our cameras are able to detect and track the robots (robot detection and tracking), the simultaneous detection of a robot by two cameras will allow the establishment of neighbourhood relationships amongst them. For example, in Figure 1 CAM1 would establish a neighbourhood relationship with CAM3 and CAM2, CAM2 with CAM1 and CAM4, *etc.* The deployment phase ends once the cameras gather their neighbourhood information. Nevertheless, these cameras will be always monitoring their neighbourhood relationships to account for changes in their pose.

At the “in use” phase, the cameras will be monitoring the environment in order to detect situations that require the presence of the robots (Call Event detection). Since the robots do not have maps of the environment, the cameras will plan routes through which the robot will navigate to get to the Call Events (distributed route planning), using their neighbourhood knowledge. Finally, the cameras will be responsible for supporting the movement of the robots along these navigation routes (remote control), for which they need to be able to detect and track the robots (robot detection and tracking). On the other hand, the robots' tasks are restricted to navigating safely towards the Call Events detected from the cameras and to provide assistance. In the next subsections, we will describe all the tasks previously mentioned.

### 5.1. Dynamic Neighbourhood Detection

Our system must guarantee the correct robot navigation towards the areas where their presence is required. We want to favour self-configuration over manual tuning, and avoid the use of prior knowledge of the environment where the robot will move. To this end, each camera of the system is capable of determining automatically which other cameras are its neighbours. This will enable the calculation of routes as sequences of cameras through which the robots will have to navigate to reach a specific area, as we will see in Sections 5.2 and 5.3.

To establish neighbourhood relationships amongst them, the cameras detect parts of their FOV that overlap with the FOV of other cameras (these overlapping areas in the FOV will be called Neighbourhood Regions from now on). Specifically, each camera divides its FOV in squared regions, and calculates the Neighbourhood Probability of each region with all the remaining cameras, *i.e.*, for a specific camera *i*, the Neighbourhood Probability of each region of the FOV of camera *i*, with respect to each camera *j*, ∀*j* ≠ *i*, is the probability of that region overlapping any part of the FOV of camera *j*.

These Neighbourhood Probabilities will be computed by detecting simultaneous events from the cameras. Whenever a camera detects a robot, it stores the region where the robot was detected and broadcasts the detection of the robot to all the other cameras. Periodically, each camera checks whether the detection of the robot is taking place simultaneously with the detection of the same robot but from any other camera. When two cameras detect a robot simultaneously, they increase the Neighbourhood Probability of the region where the robot was detected. On the contrary, when this detection does not take place simultaneously, they decrease this probability. We compute all the probability updates using a Bayesian binary filter [30].

**Figure 4.**
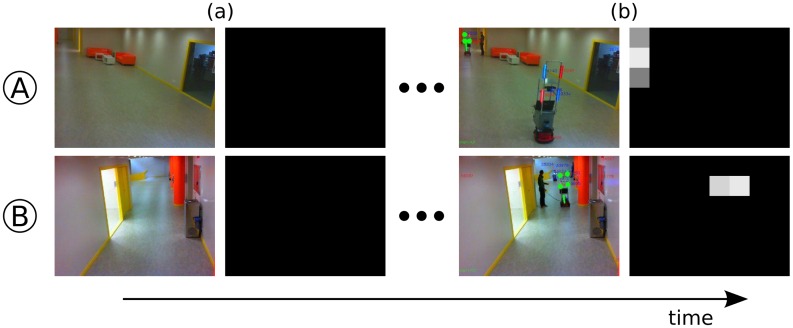
Evolution of the Neighbourhood Probability of two cameras A and B through time. Black represents a probability equal to zero, and white a probability equal to one. (**a**) Initially, the cameras assume no neighbourhood relationships. (**b**) Nevertheless, as the robot is moved around and the time elapses, the simultaneous detections allow the establishment of non-null neighbourhood values between both cameras.

Figure 4 represents the evolution of the Neighbourhood Probabilities between two cameras, A and B. Initially (Figure 4(a)), there were no simultaneous robot detections among them, so the Neighbourhood Probability was zero for all the regions. After a few common robot detections (Figure 4(b)), both cameras recognised their common regions with probability close to one. Therefore, they established a neighbourhood link, and tagged those regions as Neighbourhood Regions.

This dynamic neighbourhood detection is not only carried out during the “deployment” stage, but also during the “in use” phase, since the neighbourhood relationships can change dynamically (e.g., if some camera is moved or if it stops working). Therefore, the cameras are continuously updating this neighbourhood information to adapt to eventual changes.

### 5.2. Distributed Route Planning

A route is an ordered list of cameras through which the robot can navigate. Basically, the robot will go from the FOV of one of the cameras to the FOV of the next camera on the route, without needing metric maps of the environment. These routes will be generated as a result of local interactions amongst the cameras, without the intervention of any central agent.

**Figure 5.**
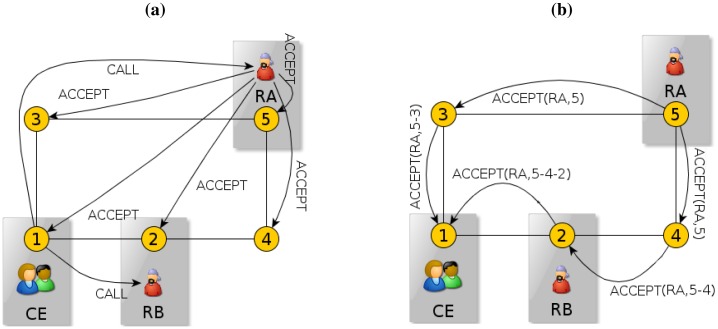
Distributed route planning procedure. Cameras (1–5) established their neighbourhood relationships, forming a network altogether. (**a**) Call event detection and call for robots. (**b**) Back-propagation process for route formation. See Section 5.2 for a detailed explanation.

We will explain the route planning process through the example shown in Figure 5. In this Figure, we represent the system as a graph, with cameras as nodes and their neighbourhood relationships as arcs. However, note that this graph is just a representation used for clarification: None of the entities in our system handle this global information. In this example, we assume that robot RA is available (willing to accept any call), while robot RB is not. We also assume that camera 1 is detecting a call event (CE), camera 2 is seeing robot RB, and camera 5 is seeing robot RA. If a camera, like camera 1 in Figure 5(a), detects a call event requiring the presence of a robot, it broadcasts a call to all the robots. If a robot is willing to attend the call, it broadcasts an acceptance to all the cameras, like robot RA does. Then, those cameras that receive this acceptance and are seeing this robot will forward it to its camera neighbours, starting a back-propagation process to create a route, as camera 5 does in Figure 5(b): Through this process, there will be a message being passed amongst the neighbouring cameras, until it reaches the camera that sees the call event. To do so, each camera that receives this message includes its identity in it, and forwards it only to its neighbours (except to those through which the message has already passed). In the figure, camera 5 includes its identity in the message and forwards it to its neighbours, camera 3 and 4, which do the same, and so on. Finally, camera 1 receives all the acceptances, so it knows that RA is willing to accept the call, and that it can follow two possible routes: 5-3-1 and 5-4-2-1. After this, this camera would select the route that involves fewer cameras (5-3-1), and inform robot RA accordingly.

It is clear after this description that the route planning does not emerge from a globally coordinated process, but from a self-organized process coming from multiple local interactions among neighbouring agents, which only handle local information.

### 5.3. Remote Control and Robot Navigation

**Figure 6.**
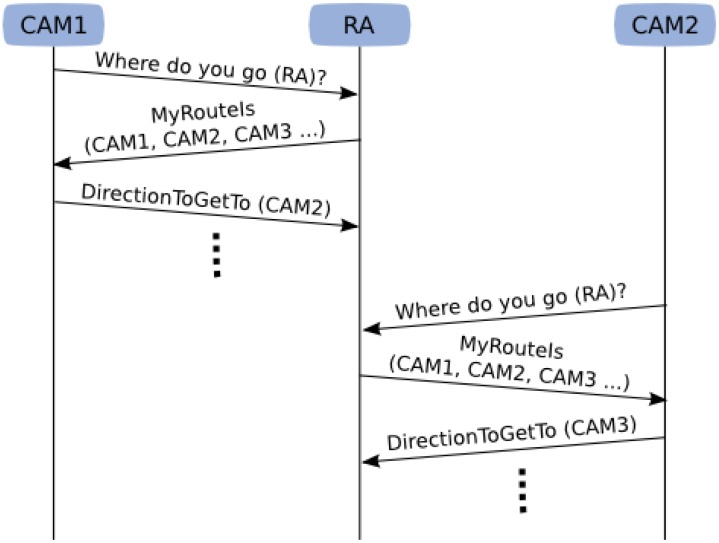
Remote control process example. First, Camera 1 (CAM1) detects the Robot A (RA) and asks it about its current route. RA answers with its route, and CAM1 corrects its movement direction towards the next camera on the route (CAM2). This process continues until RA reaches the Field of View of Camera 2 (CAM2), which will start a similar process to steer the robot towards Camera 3, and so on.

A route is a sequence of cameras. Any robot following a route must traverse the FOVs of those cameras involved in it: each camera on the route helps the robot to move towards the next Neighbourhood Region, so that the robot reaches the FOV of the next camera in the route. This remote control process is illustrated in Figure 6. First of all, if a camera sees a robot, it enquires the robot whether it is following a route, and if that is the case, for the sequence of cameras that form part of it. Then, the camera informs the robot about the direction that it should follow to get to the Neighbourhood Region shared with the next camera on the route. The camera will repeat this process (e.g., each few seconds) until the robot arrives at the next camera, which will continue guiding the robot by the same process, and so on. The control of the robot is purely reactive, updated with each new camera command. Unlike most proposals [10,11,23,31,32], we do not require our cameras to calculate the robot position in the world coordinates (just its orientation or movement direction), and hence we do not need to calibrate the cameras.

For the robot navigation, we use the classic but robust Potential Fields Method [33]: The robot moves towards the goal position commanded by the camera, which exerts an attractive force on it, while the obstacles detected by the laser scanner exert repulsive forces, so that the robot avoids colliding with them.

### 5.4. Robot Detection and Tracking

As we outline in Figure 3, the robot control and navigation and the dynamic neighbourhood detection amongst the cameras depend on the robust detection and tracking of the robots. For several reasons, we propose to mount on the robots patterns of light emitting markers (active markers) that are easy to recognize from the cameras. First of all, according to our experience, it is not possible to extract reliable features at the distances that we want our system to work (up to 20 or 30 meters), neither from the robot itself nor from passive markers mounted on it. On the other hand, we require our system to work in various illumination conditions, which can range from over illuminated spots to semi-dark places. In our experiments, we observed that neither the robot natural features nor passive markers were robust enough to cope with this (similar problems were reported by [32,34]). Moreover, regarding the task of rigid object tracking, using artificial markers tends to provide higher accuracy rates than the use of natural features [35]. Finally, the use of markers of different colours enables our system to differ among multiple robots, and thus increases the scalability of the system. Hence, we assume that the use of active markers of different colours is a very reasonable solution for our problem.

We weighed up the use of different active marker mechanisms. For instance, Cassinis *et al.* [32] built an active marker with a circular grid of high intensity red LEDs pointing down towards a mirror that reflects the light upwards to the cameras. The LEDs pulse at a given frequency that the cameras recognize to detect the robot position. The mirror's orientation, controlled by the robot, provides the robot's orientation. Similarly, Kim *et al.* [36] proposed a LED cube whose sides pulsed at different frequencies, which allowed them to measure the marker's orientation. Although both approaches are robust (especially [32]), we believe that they add unnecessary complexity for our purposes. Moreover, in our experience, the users find the light of high illumination active markers unpleasant. We also considered the use of IR LEDs [15], but their performance is very bad under natural illumination.

**Figure 7.**
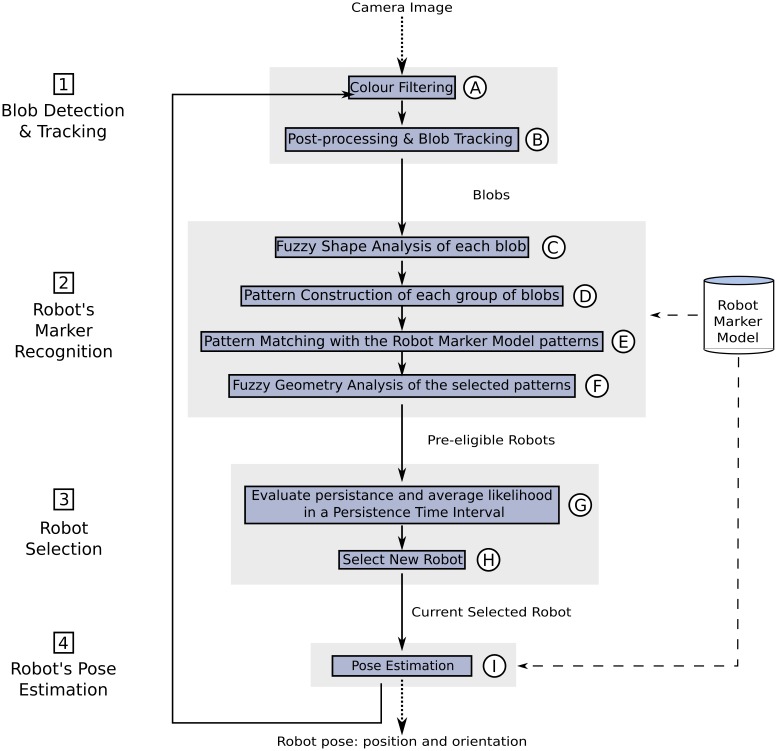
Robot detection and tracking algorithm. Continuous arrows indicate flow of execution. Dashed arrows indicate influence relationships. Dotted arrows indicate data input/output.

In the absence of a satisfactory solution to our problem in the literature, we decided to build an active marker with two red and two blue LED strips, like the one shown in Figure 2(b). Our cameras must detect this colour pattern, and measure its position and orientation, in order to retrieve the position and orientation of the robot that carries it. To do this, we use the “detection and tracking” algorithm shown in Figure 7. This algorithm performs a colour filtering to search for the colour LEDs in the camera images. Then, it seeks the robot's marker pattern in the colour filtered image, and calculates its position and orientation. We have divided this algorithm in four main stages: (1) blob detection and tracking, (2) robot's marker recognition, (3) robot detection, and (4) robot's pose estimation. In the following sections, we describe each of these stages in detail.

We would like to point out that our algorithm allows us to use more than one robot simultaneously: It is sufficient that each robot carries a marker with a different colour pattern (see the experiment in Section 6.2). However, for the sake of clarity, we will describe the algorithm with the example of the detection and tracking of a single robot that carries the marker shown in Figure 2(b).

#### 5.4.1. Stage 1: Blob Detection and Tracking

**Figure 8.**
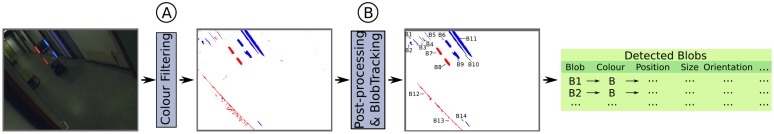
Stage 1: Blob detection and tracking.

Figure 8 shows the blob detection and tracking stage of our algorithm. In our context, a blob is a set of connected pixels of the same colour. The first step of this stage (step A, Figure 8) filters out the pixels whose colour does not match up with any of the colours of the LED strips (red and blue in this case, as in Figure 2(b)). Two kinds of pixels, and therefore blobs, will pass this filtering process: the robot blobs, and spurious blobs (pixels that match the colours of the LED strips, but are due to artificial lights, reflections of the light coming out from the robot active markers, *etc.*).

After this filtering, we assign a Kalman Filter to each blob (step B, Figure 8). Thus, we can assign an identity to each blob and track it over consecutive frames (B1, B2, B3, *etc.*, in Figure 8).

#### 5.4.2. Stage 2: Robot's marker recognition

**Figure 9.**
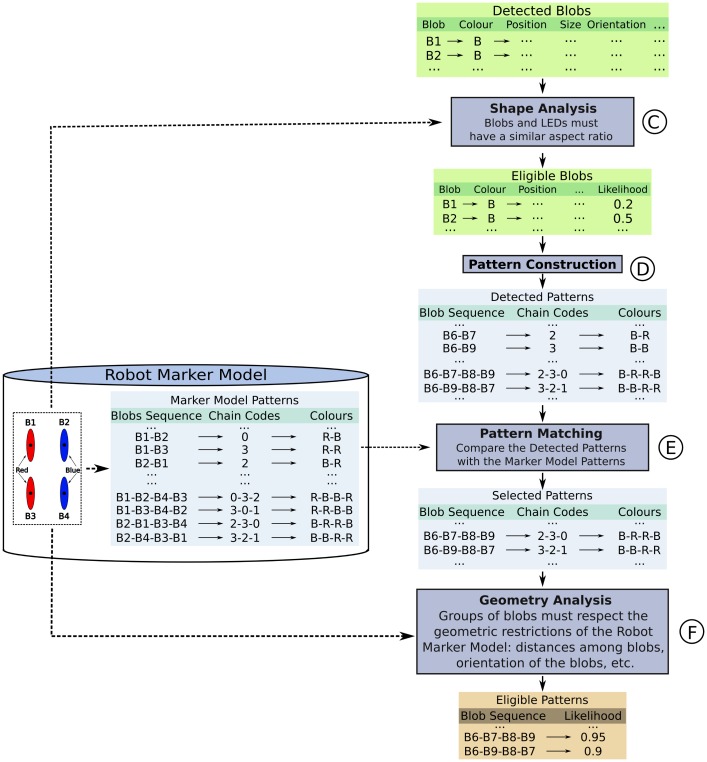
Stage 2: Robot's Marker Recognition.

In this stage, represented in Figure 9, we identify the groups of blobs that may correspond to the robot's marker LED strips. For example, in Figure 8, these blobs would be B6, B7, B8, and B9. To do this, we carry out the three stage process described in the next subsections, aimed to calculate the degree of similarity (likelihood) between each group of detected blobs and the robot marker.

##### Shape Analysis of Each Blob

First of all, we evaluate whether or not the aspect ratio (height-width ratio) of each blob is approximately equal to the aspect ratio of the LED strips of the marker (this value is six in our case). From this evaluation, we assign a likelihood to each individual blob, and only those with a non-zero likelihood will be considered in the next step (*eligible blobs*).

##### Pattern Construction and Matching

LEDs do not have univocal features, thus their blobs cannot be identified independently. Nevertheless, the group of LEDs of the marker forms a known colour pattern. To detect this pattern in the images, we can construct groups of *eligible blobs*, calculate their respective colour patterns (step D, Figure 9), and match them against the known colour pattern of the group of LEDs (step E, Figure 9). To this extent, we have chosen a pattern representation that captures (a) the blobs that form the pattern, (b) their colours, and (c) their geometrical disposition (chain codes).

**Figure 10.**
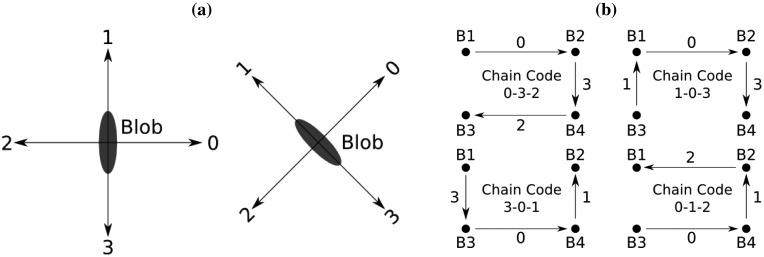
(**a**) Displacement codes of the chain code. We calculate the displacements with respect to the blob coordinates: invariance on rotations lower than ±90°. (**b**) Examples of chain codes of a set of four points. Not all possible chain codes are included.

The identification of the blobs on each pattern is straightforward, because we have already identified each blob in the blob detection and tracking step. The same applies to their colours: the colour filtering step (step 1, Figure 7) has already separated the blobs in red and blue ones. Finally, to codify the geometrical disposition of the blobs of the group, we adapt the concept of chain code [37], well known in the Computer Vision community: If, like in Figure 10(b), each blob is represented as a point, the geometry of the group of blobs can be represented by the set of sequential displacements that connect an initial blob (point) with the remaining ones. Given the shape of our marker, we allow displacements in the four directions sketched in Figure 10(a): Right, up, left and down, which can be codified as 0, 1, 2, and 3, respectively. Note that we calculate these displacements with respect to the blob coordinates, not to the image coordinates (Figure 10(a)): This makes our chain codes invariant to rotations lower than ±90°. Clearly, the points of Figure 10(b) accept several representations, depending on the election of the initial blob and on the rotation sense of the displacements (clockwise or counter-clockwise): 0-3-2, 3-0-1, 1-0-3, 0-1-2, *etc.*

At this point, we are able to construct the pattern of each group of blobs, based on the properties previously described. For instance, to construct the marker model patterns (Figure 9), we start calculating all the possible sequences of 2, 3, and 4 blobs of the model (B1-B2, B1-B2-B3, B1-B2-B3-B4, *etc.*). Then, we calculate the chain code of each sequence. For example, the sequence B1-B2-B4-B3 would be associated with the chain code 0-3-2. Finally, we construct the sequence of colours of the blob sequence. In the same example, since B1 and B3 are red, and B2 and B4 are blue, the colours for B1-B2-B4-B3 will be R-B-B-R. This codification may seem redundant, but we are just codifying all the possible colours and geometrical dispositions of the blobs, in the absence of a mechanism to identify them univocally.

We follow a similar process to construct the patterns that form the groups of eligible blobs (step D, Figure 9). In the following, we match these patterns (detected patterns in Figure 9) with the marker model patterns (step E, Figure 9). This matching process results in a list of selected patterns, which contains the patterns that form the robot groups and coincide with any of the robot marker model patterns.

##### Geometry Analysis of Each Pattern

**Figure 11.**
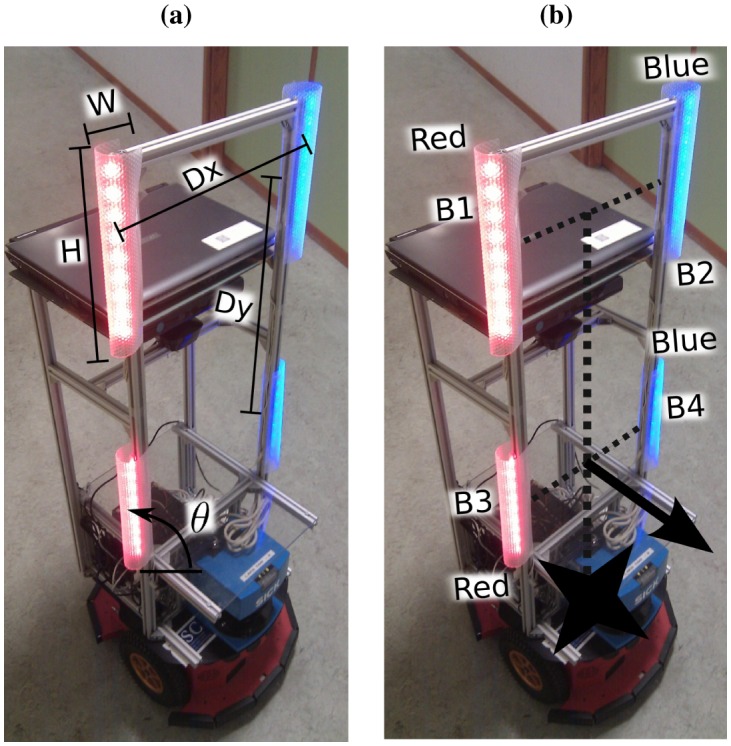
(**a**) Geometric properties taken into account in the pattern matching process: Blob width *W*, height *H*, and angle with respect to the horizontal axis *θ*, to measure parallelism among blobs, and horizontal (*D_x_*) and vertical (*D_y_*) distance among blobs. (**b**) Robot position and orientation estimation.

Finally, our algorithm measures the geometrical likelihood of the selected patterns (step F, Figure 9). This likelihood represents the degree of compliance of each selected pattern with the geometrical restrictions of the robot marker model (e.g., the distances among the blobs of the patterns must be similar to the distances among the LED strips of the marker). We compute the geometrical likelihood of each selected pattern as the product of the likelihoods of its pairs of blobs: for example, the likelihood of the group of blobs B1-B3-B4-B2 would be the product of the likelihood between B1-B3, B3-B4, and B4-B2. Then, we multiply the obtained likelihood with the likelihood of each individual blob (from the eligible blobs list, Figure 9). To calculate the likelihood of each pair of blobs, we measure the degree of compliance with the following criteria:

1. The blobs must be similar in height, width, and orientation with respect to the horizontal axis (represented as *H*, *W* and *θ* in Figure 11(a), respectively).
2. The blobs must respect certain distances among them. If the blobs are collinear (up or down displacement, like among blobs 1 and 2 in Figure 11(a)), the distance in *x* (*D_x_* in Figure 11(a)) must be approximately zero, and the distance in *y* (*D_y_* in Figure 11(a)) approximately twice the height of one of the blobs. On the contrary, if the blobs are not collinear (left or right displacement, like among blobs 1 and 3 in Figure 11(a)), *D_y_* must be approximately zero, and *D_x_* less or approximately equal to the height of one of the blobs.

Like in the case of the *chain codes* (Figure 10), all the criteria are calculated with respect to the coordinates of the blob, not with respect to the coordinates of the image (except for the blob's orientation). Hence, this likelihood measurement will be invariant to rotations.

#### 5.4.3. Stage 3: Robot Detection

So far, our algorithm is able to determine a list of blob groups that might correspond to the marker of the robot in the scene, and the degree of similarity of each group with the marker (likelihood). This list will be called the list of eligible patterns. First of all, to decide which one of these patterns corresponds to the robot that carries the marker, our algorithm discards those that do not persist over a minimum time interval (persistence time interval). Next, the algorithm calculates the average likelihood over the mentioned interval of the remaining blob groups. The persistence time interval was introduced to increase the robustness of the algorithm against spurious noises: Reflections of the marker or ambient lights, objects moving around, spurious noise that passes the colour filter, *etc.* While the robot blob groups are usually stable (high average likelihood rates over long time intervals), noise blobs are rather erratic in persistence, shape, and position (possible instantaneous likelihood peaks, but low average likelihood rates in general). On the other hand, this interval smoothens the impact of momentary drops on the likelihood of the robot blob groups.

At this point, the algorithm is able to decide whether there is a robot present in the camera images. The decision process varies depending on whether the algorithm has previously detected a robot or not. In case that the robot was detected in the previous iteration, the algorithm checks if its previously detected blob group exists in the current list of eligible patterns. If that is the case, the algorithm selects the same blob group as in the previous iteration, provided that its likelihood is higher than zero. It may also occur that some of the blobs of this group do not exist anymore. In this case, the algorithm selects the eligible pattern with the highest number of blobs in common with the group selected in the previous iteration. In following iterations, the algorithm will seek to extend this group with new blobs, to recover from the loss of the missing blobs. This ensures the stability of the robot tracking and its robustness against temporary occlusions. On the other hand, if the detected blob group detected in previous iterations disappears completely, or if the robot was not detected in the previous iteration, the algorithm simply selects the eligible pattern with the highest number of blobs. In case of a tie among two or more groups, the algorithm selects the one with the highest average likelihood over the persistence time interval.

#### 5.4.4. Stage 4: Pose Estimation

The last stage of the algorithm calculates the pose of the robot (step I, Figure 7). The robot position is calculated by projecting the centre of masses of its blobs onto the ground plane (star in Figure 11(b)). We know the real dimensions of the LED lights and their height with respect to the ground, so the projection is straightforward. With regard to the robot orientation, the algorithm first decides whether the robot is giving its front or its back to the camera, considering the chain code and the colours of its blobs. Then, the orientation direction, represented as an arrow in Figure 11(a), is calculated from the lines connecting parallel blobs. Although this does not give a high precision, it is more than enough for robot control, as we will show in our experimental results (Section 6).

## 6. Implementation and Experimental Results

We have tested our system at the Department of Electronics and Computer Science, at the University of Santiago de Compostela, Spain. The robot used was a Pioneer P3DX equipped with a SICK-LMS200 laser. On the other hand, each camera-agent used either a Unibrain Fire-i camera or a PointGrey Chameleon CMLN-13S2C with an omnidirectional lens. Both models worked at a frame rate of 15 fps and at a resolution of 640 × 480 pixels. The processing units were Intel Core 2 Duo CPUs (P8600@2.4 GHz, T5600@1.83 GHz, or T5600 Mobile P8700@2.53 GHz) with 4 GB RAM. The robot software was implemented using the Player(v-3.0.2)-Stage(v.4.0.0) platform, and for image processing (camera software) we used the OpenCV 2.2 library [38]. Regarding communications, messages were passed over an IEEE 802.11g local wireless network via UDP.

**Figure 12.**
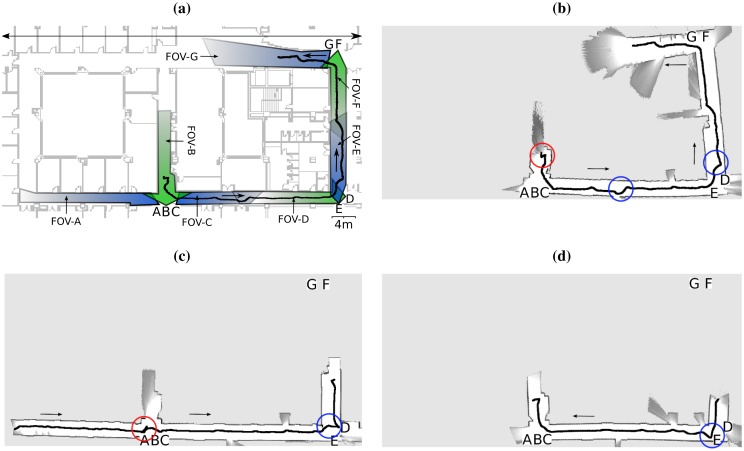
(**a**) Camera network deployed in the experiments. Each camera is represented by a letter (from A to G), and each FOV is represented by a coloured region. The trajectory represented is the same as in (b). (**b**) Robot trajectory through the route B-C-D-E-F-G (70 m long). (**c**) Robot trajectory through the route A-B-C-D-E (60 m long). (**d**) Robot trajectory through the route E-D-C-B (50 m long). We provide explanations of each trajectory in the text.

**Figure 13.**
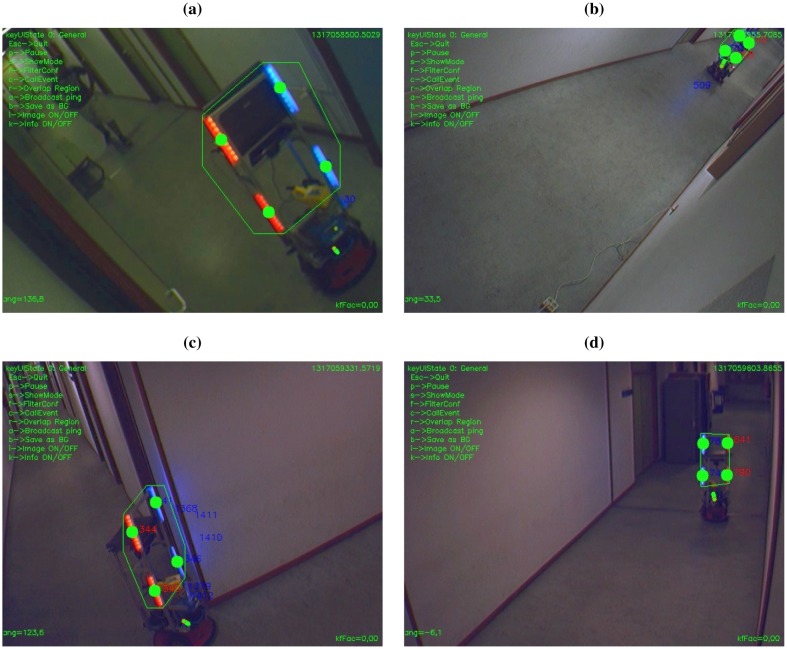
Example of camera captures from the experiment in Figure 12(c): (**a**) camera A, (**b**) camera C, (**c**) camera D, (**d**) camera E. The detected marker is rounded by a convex polygon, and each of its blobs is tagged with a circle.

We deployed the multi-agent system shown in Figure 12(a), which consists of seven camera-agents (A to G) and a robot-agent (R). Each camera covered one corridor, and each had two neighbours at most (their FOVs can be seen both in Figures 12 and 13). For instance, cameras B and C covered two perpendicular corridors, being A and C the neighbours of B, and B and D the neighbours of C. Note that this is not the only distribution that our system allows (e.g., cameras may have more than two neighbours), but the most natural one given the topology of the environment. After the deployment of the system, the cameras recognised their camera neighbours correctly. This completed the validation of the dynamic neighbourhood detection task (Section 5.1).

**Figure 14.**
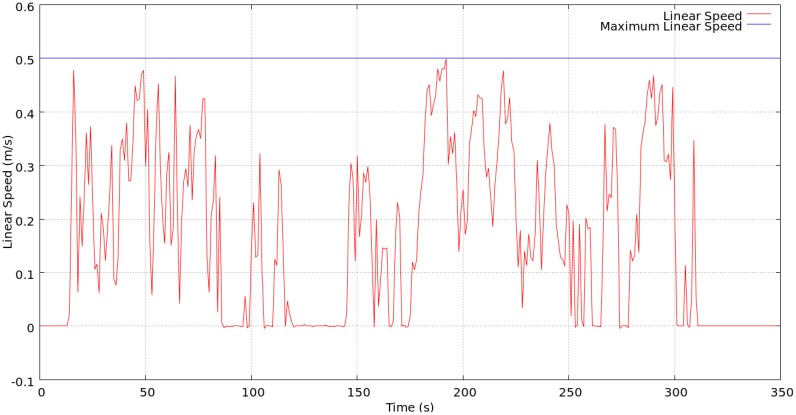
Linear speed of the robot during the experiment represented in Figure 12(c).

The rest of the experiments aimed at testing the remaining tasks of our system: distributed route planning (Section 5.2), robot detection and tracking (Section 5.4), and robot remote control and navigation (Section 5.3). Initially, there was only one camera detecting the robot, and one camera detecting a call event. In all the experiments, this last camera called the robot, and the robot accepted the call. Next, the cameras calculated the appropriate route of cameras towards the call event. Finally, the robot moved through this route supported by the cameras. We show three of the most representative robot trajectories from these experiments in Figure 12(b,c,d). In all the experiments, the robot moved at the maximum possible speed considering its weight (0.5 *m*/*s*). As an example, in Figure 14 we show the linear speed of the robot during the trajectory in Figure 12(c). The speed of the robot varies depending upon the environment and the camera commands. We obtained all these data from the robot's odometry and laser logs using the PMAP SLAM library, compatible with Player-Stage. The maps and trajectories were obtained off-line just for visualization purposes: As we have explained before, our system does not need maps of the environment.

In the first experiment (Figure 12(b)) camera G sighted the call event, started the robot call process, and triggered the route formation. Upon reception of the calculated route (B-C-D-E-F-G), the robot started the navigation towards the call event position, supported by the cameras in the route (starting from camera B) while avoiding the obstacles detected. The experiment shows that most of the trajectory is regular and most transitions among cameras are smooth. This trajectory was 70 m long.

In the second experiment (Figure 12(c)) camera D sighted the call event, and the robot navigated through the route A-B-C-E-D, starting from camera A. The trajectory was 60 m long, and as in the previous experiment, the result was satisfactory. Figure 14 represents the linear speed of the robot during this trajectory.

In both experiments, the robot movement was counter-clockwise. In the last experiment (Figure 12(d)), we tested a clockwise movement. In this case, camera B detected the call event, and the robot moved towards it through the route E-D-C-B, starting from camera E. The trajectory length was 50 m long approximately. Although the trajectories were satisfactory, we observed two kinds of anomalies that require further explanation. We tagged these anomalies in Figure 12(b,c,d) either with a red or a blue circle, depending on its type. First, red circles represent parts of the trajectory where the cameras estimated incorrectly the orientation of the robot, causing momentary navigation errors. This kind of anomaly is not common, but nevertheless it is clear that our system recovers from it very quickly. Second, blue circles represent anomalies during transitions among cameras. For instance, in the transition from C to E represented in Figure 12(b), the robot should move straight, but instead it turns slightly. This happened because the camera C provided the robot with an inaccurate orientation command to get to the FOV of camera E (Section 5.3). In general, we observed that this command becomes less accurate as the robot gets farther from the camera commanding it (C in this case) and closer to the objective area (the FOV of E, in this case). However, this problem is not critical nor common: as we can see, it did not appear in the experiments in Figure 12(c,d). On the other hand, the problem observed during the transitions from cameras E to D and vice versa is more important. During these transitions, there were time lapses when the robot navigated without guidance (blue circles). With regard to this, Figure 13(c,d) show that the FOV overlap among cameras D and E may not be sufficient, depending on the trajectory followed by the robot. These anomalies are rather harmless, but nevertheless we can correct them by increasing the FOV overlap amongst both cameras. Another option would be to use alternative systems to provide the robots with information to traverse the areas that the cameras do not cover. This would allow us to eliminate the need of FOV overlap among neighbouring cameras (Section 5.1). In this sense, we are already analysing the performance of different systems on this task [26].

We have performed many of these experiments varying the initial position of the robot and of the call event. The system proved to be robust, because the robot always arrived within a circle of 2 m around the call event area. We believe that this is sufficient for most real world applications where our system could be used. For example, this would suffice if the robot is meant to approach groups of people to offer them information, or to navigate towards areas where an anomaly is detected. On the other hand, in all the experiments that we have carried out, the success of the robot was 100%. This success rate depends on the number of the cameras and the dimensions of the space they cover. For this reason, in the next section we perform a careful analysis of the maximum recommended working range of each camera, and some other aspects that are relevant to guarantee such a high success rate. In future works, we plan to measure how this success rate varies according to factors such as the number of the people in the environment, the length of the trajectory, *etc.*

### 6.1. Robot Detection and Tracking

The robot detection and tracking algorithm is a critical part of the system, and has been explained thoroughly in Section 5.4. We have considered it appropriate to undertake a quantitative analysis of its performance, which we detail in this section. To this extent, we have constructed a dataset from the video recorded by each of the five cameras of the experiment in Figure 13(c). In this experiment, camera B recorded the robot at a distance up to 4 m (approximately), E up to 15 m, A and C up to 30 m, and D up to 50 m. The cameras recorded video at 15 fps during 14 minutes, making a total of 12564 frames in our dataset.

We have executed our algorithm on this dataset with different parameter variations to test their impact on the performance of the algorithm. Particularly, we have considered variations on:

- Colour Filters. To detect the marker of the robot, we need to detect its colours first (see Section 5.4.1). We define each colour that has to be detected as an interval in the HSV colour space. To detect the colours of the marker, we filter out every pixel whose colour is out of the desired intervals. We observed that the illumination conditions affect the S and V colour components very strongly, thus their corresponding intervals have to be adjusted for each environment. A careful adjustment is prohibitive if we want to deploy our robots quickly, but a coarse one may let undesired colours pass the filtering stage. For these reasons, we assessed the performance of the algorithm with two colour filters: (1) one with carefully adjusted intervals, and (2) another with coarsely adjusted intervals.
- Persistence Time Interval. This refers to the number of consecutive frames in which the robot marker has to be detected to consider that there is a robot in the FOV of the camera (see Section 5.4.3). We analysed two scenarios to measure the impact of this interval: (1) interval length equal to zero (no interval), (2) interval length equal to 10 frames (0.66 s).

For each frame, our algorithm tells us whether it detected the robot, and if that is the case, its position. To analyse the results, we classified each frame into one of the following five classes (confusion matrix in Table 1):

- *True Positive* (*TP*): If there was a robot in the scene and the camera detected it. The higher the better.
- *False Positive*—*Robot in Scene* (*FP_RS_*): If there was a robot in the scene and the camera detected another element. The lower the better.
- *False Negative* (*FN*): If there was a robot in the scene and the camera did not detect it. The lower the better.
- *False Positive*—*No Robot in Scene* (*FP_NRS_*): If there was no robot in the scene but the camera detected one. The lower the better.
- *True Negative* (*TN*): If there was no robot in the scene and the camera did not detect any robot. The higher the better.

We divided the False Positives in two different groups to have a separate measure of the noise caused by the markers' lights reflections (which commonly causes the *FP_RS_* rate) from the rest of the noise (which commonly causes the *FP_NRS_* rate).

According to our experience, the accuracy of the remote control of the robot from the cameras drops significantly at distances higher than 20 or 30 m. Moreover, at this distance, the marker is too small to be detected accurately. Therefore, we usually restrict the effective coverage of the cameras to distances below this range. For this reason, to analyse the results, we considered two different levels of requirement regarding the maximum distance at which our algorithm must detect the robot: (1) arbitrarily large distances, and (2) distances below 20 m. We are mostly interested in this last case.

Table 2 shows the results that we have obtained in this analysis. From these results, we can draw the following conclusions. First of all, Table 2 shows that the average *FP* rates are low, and the average *TN* rates are high. This ensures that when the algorithm detects a robot, this detection is usually correct. This is very adequate for our needs, because an incorrect robot detection may result in an incorrect command sent to the robot, and thus in a wrong robot navigation, at least until the next command. In the same column, we see that the average *TP* and *FN* rates are not ideal, but acceptable for our purposes: If a camera fails to detect a robot occasionally, our system will still be able to support the robot navigation, because the acquisition rate of each camera is high with respect to the speed of the robot. In fact, it is enough if the cameras are able to detect and send a command to the robot every few seconds. On the other hand, we observe that the results of the algorithm are the best when we use carefully adjusted colour filters. However, to tune them in every camera at deployment time would be prohibitive. Fortunately, we also observe that we obtain similar results if we use a persistence time interval of 10 frames, even with coarsely adjusted colour filters. Specifically, this improves the *FP* and *TN* rates, which are the most important for us. Therefore, we can adjust the colours filters coarsely, which can be done very quickly, and still achieve a great robustness on the detection and tracking of the robot. Finally, as it was expected, the overall results of the algorithm improve when we do not require the algorithm to detect the robot at distances greater than 20 m. The most typical scenario in real deployments (like experiments in Section 6) is remarked in boldface.

We have also used the following metrics to measure the classifier performance [39]: Positive Predictive Value or Precision (*PPV*, Equation (1)), Negative Predictive Value (*NPV*, Equation (2)), True Positive Rate or Sensitivity/Recall (*TPR*, Equation (3)), and Matthews Correlation Coefficient (*MCC*, Equation (4)). *PPV*, *NPV*, and *TPR* range in the [0, 1] interval (the higher, the better), and the Matthews Correlation Coefficient (*MCC*) ranges in [−1, 1] (−1 representing an inverse prediction, 0 an average random prediction and +1 a perfect prediction).

(1) $$\text{PPV}=\frac{\text{TP}}{\text{TP}+{\text{FP}}_{\text{RS}}+{\text{FP}}_{\text{NRS}}}$$

(2) $$\text{NPV}=\frac{\text{TN}}{\text{TN}+\text{FN}}$$

(3) $$\text{TPR}=\frac{\text{TP}}{\text{TP}+\text{FN}}$$

(4) $$\text{MCC}=\frac{\text{TP}\cdot \text{TN}-({\text{FP}}_{\text{RS}}+{\text{FP}}_{\text{NRS}})\cdot \text{FN}}{\left(\text{TP}+{\text{FP}}_{\text{RS}}+{\text{FP}}_{\text{NRS}})(\text{TP}+\text{FN})(\text{TN}+{\text{FP}}_{\text{RS}}+{\text{FP}}_{\text{NRS}})(\text{TN}+\text{FN}\right)}$$

For the reasons already mentioned, the robot control problem requires to maximize the *PPV* rate (proportion of frames where the robot is detected and the detection is correct), and maintain to an acceptable rate the *NPV* (proportion of frames where the robot is not detected and there is no robot) and the *TPR* values (proportion of frames where there is a robot and is correctly detected). The results in Table 2 confirm that our algorithm is very well suited to this problem. Moreover, the *MCC* values, which measure the quality of the classifications, confirm the good performance of the proposed classifier.

Summarizing, we conclude that our algorithm is very robust to be used in the context of robot control. We have also observed that the use of the persistence time interval ensures the robustness of the algorithm even with coarsely adjusted colour filters. This guarantees that we will be able to deploy our system in different environments in a fast and easy manner. Considering these results, we think that it is important to highlight that in all the experiments of Section 6 we have used the same colour filters (coarsely adjusted), for all the cameras, and regardless of the illumination conditions.

### 6.2. Scalability with the Number of Robots

The architecture of our system allows the management of multiple robots naturally. Each agent has a unique ID, which allows it to communicate and coordinate with the rest of agents. Obviously, the cameras must identify each robot univocally to assign it an ID, so our robots carry active markers with unique colour patterns. Considering this, the weakest part of our system concerning scalability lies in the proper identification of each robot from the cameras.

**Figure 15.**
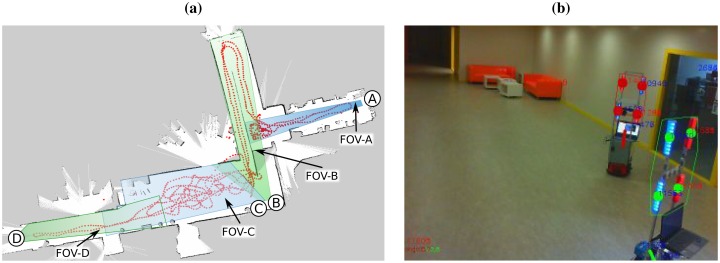
Deployment of our self-organised multi-agent network with two robots working in the environment. (**a**) FOV of each camera, and trajectory of one of the robots. (**b**) Snapshot of both robots taken from camera C.

We have performed an experiment to test our robot detection and tracking algorithm with more than one robot. To this extent, we have deployed four cameras, and we moved two robots around (Figure 15(a)). Figure 15(b) shows both robots seen from one of the cameras.

Table 3 shows the results of the experiment. On the one hand, the *TP*, *TN*, *FP_RS_*, *FP_NRS_*, and *FN* rates are similar to those obtained with one robot (Section 6.1). On the other hand, the misclassification rate (*MISCL*) indicates that our algorithm almost never confuses one robot with the other. This ensures that increasing the number of robots to be detected does not degrade the performance of the algorithm.

Finally, Table 4 shows the processing time per image of our algorithm using a modern computer (Intel i7-2670QM CPU @ 2.20 GHz, 4 GB RAM). We can extract three main conclusions. First, it takes around 16 *ms* to detect a robot on each image. This time is very low, taking into account that: (1) our cameras provide one image each 66 *ms* (acquisition rate of 15 *fps*), and (2) they send commands to the robots every 2 seconds at most. Second, the computational time does not increase when executing more than one camera concurrently, as expected of a multi-core processor. Finally, the processing time scales linearly with the number of robots to be detected, each robot increasing this time in 16 *ms* (e.g., 2 robots require 32 *ms*). We would like to point out that the current processing time is good enough for our requirements, but there is still a lot of room for improvement: code optimization, parallelization, use of GPU, *etc.* For example, right now each camera computes the detection of all the robots in the same CPU core, but it could instead compute the detection of each robot in a different core.

All in all, our system is able to handle more than one robot, because: (1) the software architecture allows it, (2) the cameras can identify each robot univocally, and (3) in the worst case, the computational time required scales linearly with the number of robots.

## 7. Summary, Conclusions, and Future Work

In this paper, we described a robotic system intended to deal with two problems that are preventing cutting edge service robots to work out of the research centres: The cost of their deployment in new environments, and the poor perception of the users on the quality of their services. On the one hand, to be cost-effective, the deployment of the robotic systems should be automatic, fast and easy, but in practice it usually requires several days of adaptation to the environment conditions. On the other hand, the perception of the users on the quality of the robot services is tightly related to the ability of the robots to perceive their needs and to show initiative to attend them, regardless of where they happen in the environment. To tackle these problems, we have combined robotics, ambient intelligence and ubiquitous computing technologies to propose a system fast and easy to deploy, which enables our robots to perceive and attend users' needs anywhere in the environment.

Our system consists of a distributed multi-agent network formed by two kinds of agents: intelligent cameras that can be easily spread out on the environment, and autonomous robots navigating in it. Each camera can discover its camera neighbours by detecting simultaneous events in their Field of View. This is a self-organized and decentralized process that automatizes the deployment and speeds up the start-up of the system. The cameras serve two main purposes. On one side, they detect situations that require the presence of the robots. This increases the range of perception of the robots and enhances the opinion of the people on their pro-activity On the other, the cameras support the robot navigation in absence of a map of the environment. This is done by means of two self-organized processes: (1) the cameras calculate the routes of cameras through which the robot can move, and (2) each camera helps the robot to get to the next camera on the route. All of this results in a robust, flexible and scalable system.

We demonstrated in real world experiments that our robots are able to attend to events detected by the cameras, regardless of the positions of the robots and the events. Given its importance for the performance of our system, we have also proposed a method for the detection and tracking of our robots. Experimental results showed its robustness in the context of robot control, even in the presence of noise and under different illumination conditions.

As part of our ongoing research, we will apply techniques [40,41] to infer camera neighbourhood relationships from the trajectories of people in the environment, even if the cameras do not detect these people simultaneously. On the other hand, we are integrating mapping and localization strategies in our system to increase the robustness of the robot navigation in areas poorly covered by the cameras. Finally, we are working on the recognition of scenes that require the presence of our robots: groups of people standing still for long periods of time, people waving at the cameras, *etc.*

## Figures and Tables

**Table 1.** Confusion matrix used to classify the robot detection and tracking algorithm results.

			Is there a robot?	
			Yes	No
Detects	Robot	The robot is the same as the real robot	*TP*	*FP_NRS_*
		The robot is different than the real robot	*FP_RS_*	
	Nothing		*FN*	*TN*

**Table 2.** Classification results of the detection and tracking algorithm. We have analysed eight different scenarios by varying: MaxDist (maximum distance at which we require the cameras to detect the robot), ColFilt (whether the colour filters have been coarsely or carefully adjusted), and Int (time persistence interval). The typical configuration that we use on robot control experiments (like those on Section 6) is remarked in boldface. The acronyms are: *TP* (True Positives), *TN* (True Negatives), *FP-RS* (False Positive—Robot in Scene), *FP-NRS* (False Positive—Not Robot in Scene), *FN* (False Negatives), *PPV* (Positive Predictive Value or Precision), Negative Predictive Value (NPV), *TPR* (True Positive Rate or Recall), *MCC* (Matthews Correlation Coefficient).

	MaxDist = ∞				MaxDist = 20*m*				Avg
	
	ColFilt = Coarse		ColFilt = Careful		ColFilt = Coarse		ColFilt = Careful		
	
	Int = 0	Int = 10	Int = 0	Int = 10	Int = 0	Int = 10	Int = 0	Int = 10	
*TP*	0.528	0.451	0.609	0.516	0.760	**0.614**	0.872	0.764	0.639
*TN*	0.958	0.999	1.000	1.000	0.946	**0.999**	1.000	1.000	0.988
*FP_RS_*	0.238	0.007	0.018	0.006	0.132	**0.003**	0.028	0.009	0.055
*FP_N RS_*	0.042	0.001	0.000	0.000	0.054	**0.001**	0.000	0.000	0.012
*FN*	0.234	0.543	0.373	0.479	0.108	**0.383**	0.101	0.228	0.306

*PPV*	0.627	0.983	0.971	0.989	0.725	**0.992**	0.969	0.989	0.906
*NPV*	0.881	0.769	0.829	0.790	0.962	**0.893**	0.966	0.926	0.877
*TPR*	0.693	0.454	0.620	0.519	0.876	**0.616**	0.897	0.770	0.680
*MCC*	0.485	0.632	0.700	0.666	0.713	**0.717**	0.879	0.804	0.699

**Table 3.** Classification results of the detection and tracking algorithm with two robots. *MISCL* (misclassification rate) is the percentage of frames where the algorithm confuses one robot with the other. The rest of the acronyms have been defined in Section 6.1.

	*TP*	*TN*	*FP_RS_*	*FP_NRS_*	*FN*	*MISCL*
*Robot*1	0.781	0.995	0.031	0.005	0.18	0.004
*Robot*2	0.821	0.996	0.283	0.004	0.151	0.009

**Table 4.** Execution times of the robot detection and tracking algorithm. *μ* is the average processing time per image (in milliseconds), and *σ* is the standard deviation (in milliseconds). NumRobots refers to the number of different robots that the algorithm tries to detect. NumCams refers to the number of camera-agents executed on a single computer (concurrently).

	NumRobots = 1			NumRobots = 2		
	
	NumCams = 1	NumCams = 2		NumCams = 1	NumCams = 2	
			
	Cam1	Cam1	Cam2	Cam1	Cam1	Cam2
*μ*(*ms*)	15.66	16.60	16.31	32.57	32.63	32.57
*σ*(*ms*)	1.37	1.49	1.67	3.27	3.24	3.27
